# Structural insights into RNA cleavage by a novel family of bacterial RNases

**DOI:** 10.21203/rs.3.rs-3788707/v1

**Published:** 2023-12-27

**Authors:** Ruoxi Wu, Sarah Barnes, Heather Dahlin, Susmita Khamrui, Shakti Ingle, Yufei Xiang, Yi Shi, David Bechhofer, Michael Lazarus

**Affiliations:** Icahn School of Medicine at Mount Sinai; Icahn School of Medicine at Mount Sinai; Icahn School of Medicine at Mount Sinai; Icahn School of Medicine at Mount Sinai; Icahn School of Medicine at Mount Sinai; Icahn School of Medicine at Mount Sinai; Icahn School of Medicine at Mount Sinai; Icahn School of Medicine at Mount Sinai; Icahn School of Medicine at Mount Sinai

## Abstract

Processing of RNA is a key regulatory mechanism for all living systems. We recently discovered a novel family of endoribonucleases that is conserved across all bacteria. Here, using crystallography, cryo-EM microscopy, biochemical, biophysical, and mass spectrometry techniques, we are able to shed light on a novel RNA cleavage mechanism in bacteria. We show that YicC, the prototypical member of this family, forms a hexameric channel that closes down on a 26-mer RNA substrate, and find that it cleaves across an RNA hairpin to generate several short fragments.

## Introduction

Dozens of bacterial ribonucleases that are involved in the processing and turnover of all types of RNA have been discovered in recent years, with the surprising finding that the complement of RNases in different organisms can vary greatly. For example, for the model Gram-positive *B. subtilis* and Gram-negative *E. coli* bacteria, nine of the known *B. subtilis* RNases are also found in *E. coli*, whereas twelve other *B. subtilis* RNases are not found in *E. coli* ^1^. There are typically redundant functions for several RNases of a particular class (e.g., endonuclease, 3’ exonuclease), such that the deletion of an RNase-encoding gene may not show a strong phenotype.

Recently, our laboratory discovered a novel endoribonuclease in *B. subtilis*, YloC, in a strain that was deleted for genes encoding the four known 3’ exoribonucleases ^2^. YloC is a divalent cation-dependent hexameric endonuclease whose in vivo function is unknown. The identity of YloC as an endoribonuclease was a surprise, as one could not predict this from its sequence, which has little in common with known ribonucleases. YloC belongs to the so-called YicC family of RNases, named after the *E. coli* homologue, with which it shares 30% identical residues. YloC and YicC catalyze the same endonuclease reaction *in vitro*. YicC family members have been implicated in several areas of bacterial physiology, including stress response ^3^, sporulation ^4^, iron regulation ^5^, and DNA repair ^6^. YicC-like proteins are widespread in bacterial species and have highly conserved N-terminal and C-terminal domains. The C-terminal domain is characterized as “DUF1732,” i.e., a domain of unknown function without a reliable annotation in the Pfam database ^7^, and it is in the top 20 of DUFs that are present in 500 or more species ^8^.

While we were preparing this manuscript, Huang *et al*. published their findings on the structural and biochemical characterization of *E. coli* YicC ^9^. These authors determined a low resolution YicC crystal structure (4.05 Å), and confirmed that the protein forms a hexamer. Using RNA oligonucleotide substrates (oligos) to assess RNase activity, Huang *et al*. identified a GUG sequence as a YicC target, with cleavage occurring after the U residue. The nature of the nucleotide immediately upstream of the GUG motif was also reported to contribute to cleavage specificity.

In this report, we determined the crystal structure of YicC to 2.8 Å, providing a detailed view of the apoprotein. We then also determined the cryo-EM structure of YicC bound to a 26-mer RNA substrate that forms a hairpin in the active site. RNase assays using the 26-mer RNA oligo identified a single major and a single minor site for YicC cleavage, which were located on either side of a double-stranded stem, as identified by mass spectrometry analysis. Multiple YicC mutant proteins were assayed as well, suggesting key residues required for RNA binding and catalysis. Based on the clamshell-like structure of the open and closed conformations of YicC, we propose to name this ribonuclease RNase CS.

## Results

### Crystal structure of apo YicC

**Figure 1:**
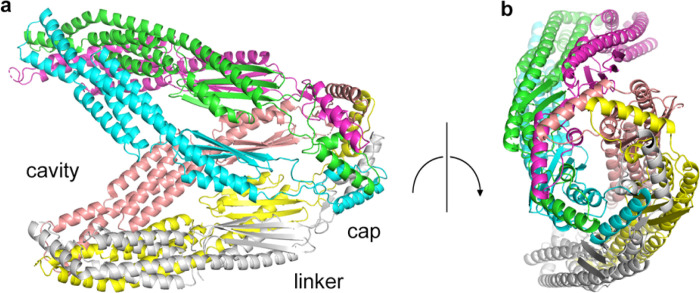
Crystal structure of apo YicC. **a,** side view of YicC hexameric complex, with regions of the protein indicated. **b,** end view of the complex, focusing on the hexameric cap. The structures are colored according to chain.

In order to gain insight on the function and mechanism of this new family of endoribnucleases, we undertook structural studies. We were unable to obtain crystals of YloC, but we obtained crystals of YicC that diffracted to 2.8 Å anisotropically. We then solved the structure using the AlphaFold predicted model of YicC as a search model and refined the structure to good Rfree and stereochemical statistics (Table 1). As we had previously predicted, the protein is a hexamer with six copies in the asymmetric unit, forming a twisted clamshell open structure (Fig. 1A). The protein complex is oligomerized by a cap domain that occurs in the middle of the protein sequence but forms a tight hexamer at the end of the protein (Fig. 1B). The cap is then connected to the rest of the protein by a flexible linker region. The protein opens up into a pincer structure formed by a dimer of symmetric trimers, with a cavity large enough to accommodate an RNA sequence. We also noticed several density areas that formed along a series of arginine residues in the cavity. We assigned these to sulfates from the crystallization condition but hypothesize that they are mimicking the phosphates from the RNA backbone that need to be bound in the active site (Figure S1). The large number of basic residues could help bind RNAs in the cavity.

### Cryo-electron microscopy structure of YicC bound to a 26-mer RNA

**Figure 2:**
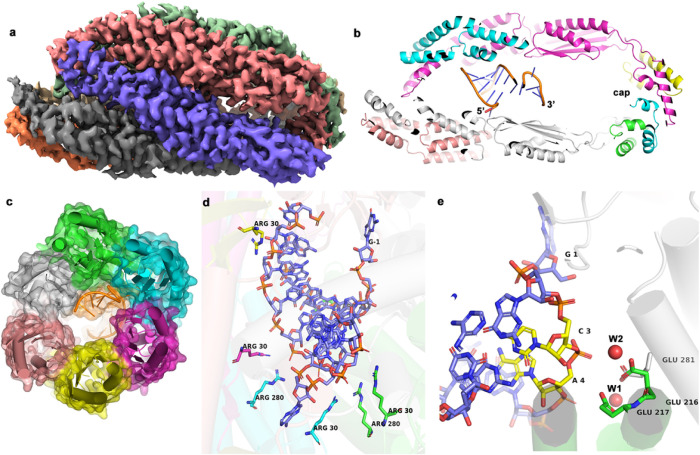
Cryo-EM structure of YicC bound to substrate RNA. **a,** electron density from cryo-EM structure, colored by chains. The map is shown in the same orientation as the apo structure in Fig. 1a. **b,** sectional view of overall structure, illustrating RNA bound in the cavity. Ends of RNA are labeled. **c,** end view of complex, with surface view of YicC colored by chain. **d,** arginine resides help bind the RNA backbone. The arginine sidechains are colored according to chain. Selected arginines are shown that are in proximity of the phosphate backbone. **e,** catalytic site of YicC. The proposed cleavage bases, C3 and A4 are colored in yellow. Water molecules in structure are shown as red spheres, W1 and W2. Essential glutamates are shown, colored according to chain.

**Figure 3:**
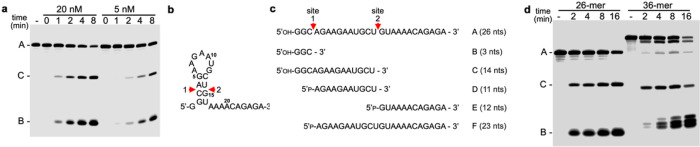
Fluorescent assay of RNA cleavage. **a**, Time course of YicC activity. RNA concentration was 600 nM; YicC protein concentration was as indicated above each set of reactions. Reactions were incubated at 37°C and aliquots were removed at times indicated above each lane. Control lane (−), no YicC protein added. Migration of full-length RNA oligo (A) and cleavage products (C and B) indicated at left. **b,** Sequence and secondary structure of 26-mer RNA used in the cryo-EM study. The same 26-mer, but with a 5’-IR800 fluorophore, was used for cleavage assays. Two sites of YicC cleavage are shown. **c,** Predicted cleavage fragments of 26-mer RNA. Fragments C, D, and E were confirmed by mass spectroscopy. **d,** YicC cleavage of 26-mer and 36-mer. RNA concentration in each case was 600 nM; YicC concentration was 20 nM for the 26-mer reaction and 100 nM for the 36-mer reaction.

We then sought to obtain structural information on how YicC binds RNA. Using a 26-mer oligonucleotide (DHB2051, Table S1), we co-purified YicC on gel filtration with RNA bound, in the absence of Mg^2+^ to prevent cleavage. We froze grids and collected data on the complex using a Krios microscope. After processing the data, we obtained density for the complex at 3.22 Å resolution, including good density for the RNA (Table 2 and Figures S2-S4). To our surprise, the predominant species was a closed complex that resembled a barrel (Fig. 2A). The protein undergoes a significant conformational change to achieve the closed position, with the closest points moving over 50Å as the trimers close in on each other. The closed RNA complex is a pseudo dimer, with one trimer recessed and the other trimer extending outward. This asymmetry allows the hexameric complex to present different sets of residues on either side of the RNA. The RNA binds as a hairpin in the middle of the barrel (Fig. 2B and Fig. 3A). The RNA occupies the cavity formed by the closed structure, with the 5’ helical portion aligning parallel to the barrel of the protein and the 3’ end extending towards the cap (Fig. 2C). On one side of the hairpin are the arginine residues we observed in the apo structure, specifically Arg30 and Arg280, that create a positively charged surface along the cavity (Fig. 2D). We observed two spheres for density in proximity to several glutamate residues (Fig. 2E) near positions 3 and 4 of the RNA. We assigned these to water molecules, but believe they could be the sites of magnesium ions in a catalytic complex, since we know the ribonuclease activity is divalent-cation dependent. Based on our structures, we predicted that the glutamates near the 5’ proximal cleavage site would be essential for catalysis, likely through coordinating magnesium. This would be reminiscent of the double metal-ion mechanism of RNase H ^10, 11^. RNase III also cleaves hairpin RNAs using a double Mg^2+^ catalytic site.^12, 13^

### Fluorescent assay for cleavage of RNA

In order to monitor cleavage of the reaction, we used an IR-fluorescent labeled version of the RNA that was in our cryo-EM structure (DHB2150, Table S1). With a 5’ label, we were able to follow cleavage of the RNA oligonucleotide and ran a short time course (Fig. 3B). We observed only two bands that increase over time, suggesting that the enzyme cleaves at two places in the RNA. The smaller cleavage product B is the major product but the larger product C seen on the gel also increases over time. In this assay we can only observe fragments that contain the 5’ labeled end, but cleavage at two sites suggests that there could be as many as five fragments total generated by the enzyme (Fig. 3C, Fragments B-F).

The initial assay used to detect ribonuclease activity in *B. subtilis* extracts^2^ employed an IR-fluorescent labeled 36-mer (DHB1879, Table S1), with a different sequence from the 26-mer used in structural studies described above. We assayed cleavage of this RNA, side-by-side with cleavage of the 26-mer (Fig. 3D). YicC was far less active on the 36-mer, and five times as much protein was used to see significant accumulation of cleavage products. Digestion of the 36-mer gave a defined larger cleavage product, which was slightly smaller than 26-mer cleavage product C, and a group of three small products, which were slightly larger than 26-mer product B. Thus, for two different RNA substrates, we observed cleavage proximal to the 5’ end and a short distance downstream.

We reported previously that YloC of *B. subtilis* and YicC of *E. coli* cleave a particular RNA substrate at the same positions. The homology between the YicC and YloC protein sequences is relatively high: 30.2% identity and 52.9% similarity. We noticed that the homology between the YicC-family protein from *Borrelia burgdorferi (B. burgdorferi),* the causative agent of Lyme disease, and *E. coli* YicC was considerably less: 25.6% identity and 48.2% similarity. It was of interest to determine whether the *B. burgdorferi* YicC family protein also had a similar ribonuclease activity. We therefore analyzed the cleavage patterns for YicC, YloC, and *B. burgdorferi* YicC (Fig. 3D). Indeed, the *B. burgdorferi* YicC protein also showed endoribonuclease activity. However, the B fragment appeared to migrate slightly slower and there were two fragments of the C size.

### Mass spectrometry analysis of cleavage

We were unable to determine the exact sizes of the fragments observed in the gel assay of YicC cleavage products. In addition, we could only observe the 5’ cleavage products, due to the fluorescent label on this end. Therefore, we turned to mass spectrometry to monitor the unlabeled cleavage reaction, with the same substrate that was used in our cryo-EM structure. There are five potential cleavage fragments based on the two observed cleavage events (Fig. 3C). In order to analyze the samples by mass spectrometry, the reaction products were purified by phenol-chloroform extraction and ethanol precipitation. To assess the precipitation of small RNA fragments, we performed a reaction in parallel that contained the unlabeled 26-mer RNA and included a trace amount of 5’-end labeled 26-mer RNA. As could be expected, we found that the 5’-terminal 3-nt fragment B could not be precipitated (data not shown). After we analyzed the purified reaction by mass spectrometry, we were able to observe three major products: the 14-mer from 1–14 (Fragment C), the 11-mer from 4–14 (Fragment D), and the 12-mer from 15–26 (Fragment E). The mass spectra had the precise masses and fragmentation patterns corresponding to the RNA sequence and predicted cleavage sites (Supplementary Figure S5). These data are consistent with cleavage between residues 3 and 4 and residues 14 and 15 (Fig. 3A). Fragment F was not detected by mass spectrometry, either because the fragment cannot easily be detected or because a cleavage at site 1 is immediately followed by cleavage at site 2.

### Analysis of mutants of YicC

**Figure 4:**
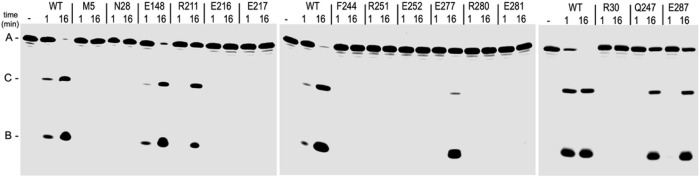
Screening of YicC residues required for cleavage. 26-mer RNA concentration was 600 nM; YicC protein concentration was 20 nM. Reactions were incubated at 37°C and aliquots were removed at 1 min and 16 min. The YicC residue that was mutated to alanine is indicated for each mutant protein. Control lane (−), no YicC protein added. Migration of full-length RNA oligo (A) and cleavage products (C and B) indicated at left.

To test the involvement of specific YicC residues, targeted mutagenesis was performed; the targeted residue was changed in each case to alanine. Fifteen mutant proteins were expressed and purified, and RNase activity was assessed using the gel assay, as in Fig. 3A, with aliquots removed after 1 min and 16 min. The data in Fig. 4 show that wild-type YicC cleaves a significant amount of the RNA oligo after 1 min and almost 100% of the substrate at 16 min. A similar pattern was seen in four of the mutants: E148, Q247, E277, and E287 – indicating that these residues are not involved in RNA binding or catalysis and are not essential for protein folding. Ten of the mutants showed no cleavage activity in this assay: four of these were changes of glutamate residues (E216, E217, E252, E281) which we hypothesized were involved in catalysis, and three of these were changes of arginine residues (R30, R251, R280), two of which we hypothesized earlier were involved in RNA binding (Figure S1). The lack of activity for these mutants supports these hypotheses. The other three residues – M5, N28, and F244 – were targeted based on their conservation across species and may be involved in helping to position the catalytic and binding residues. Interestingly, the R211 mutant gave some RNase activity, but it appeared that there was more of fragment C than fragment B. To confirm this, a time course was performed, comparing wild-type YicC to the R211A mutant (Supplementary Fig. S6). The result showed that, indeed, the ratio of cleavage products for the R211 mutant was inverted with respect to wild type, i.e., there was about half as much fragment B as there was fragment C. This may suggest that binding of the RNA and presentation to the catalytic site is affected by the R211 mutation.

**Figure 5:**
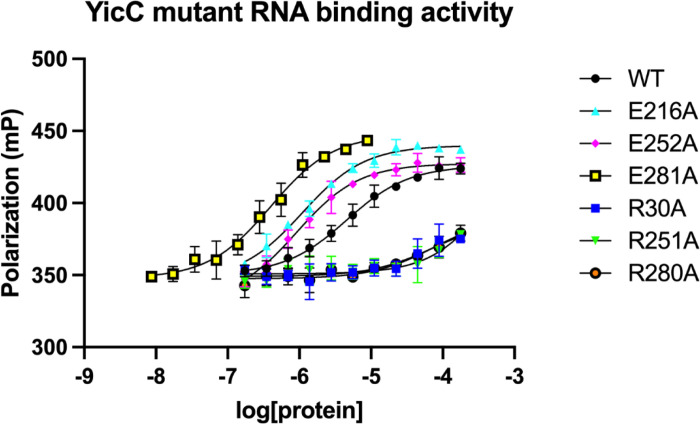
Analysis of YicC mutant binding to RNA by fluorescence anisotropy. **Binding constants were measured** using a fixed concentration of 5’-Cy3 labeled RNA 36-mer and varied concentrations of wild-type or mutant YicC protein. Fluorescent polarization was measured on a multimode plate reader. The binding constants are shown in Table S2. n=3 for each data point, shown with error bars for standard deviation.

To demonstrate whether YicC mutant proteins that showed no RNase activity were defective for catalysis or for RNA binding, we performed fluorescence anisotropy experiments to measure the K_d_ of RNA-protein binding for three glutamate and three arginine mutants. The RNA substrate in this case was a 5’-Cy3 end-labeled 36-mer RNA. This RNA substrate gave a 5 µM K_d_ value for the wild-type protein. Remarkably, all three arginine mutants showed greatly impaired binding, while the three glutamate mutants showed slightly better or even much better binding (Fig. 5 and Table S2). Thus, the loss of RNase activity for the glutamate mutants is due to an effect on catalysis, while the loss of RNase activity for the arginine mutants is due to deficient RNA binding. Specifically, the cluster of glutamate residues, including E216 (and E217), E251, and E281, was likely involved in catalysis by coordination of Mg^2+^ ions (where we observed water molecules in our cryo-EM structure), while the cluster of arginine residues, including R30, R251, and R280, was likely involved in RNA binding by electrostatic interactions.

## Discussion

That YicC specifies endoribonuclease activity could not be predicted from its primary sequence, as there is little homology to any of the known ribonucleases. Solution of the YicC apoprotein and YicC-RNA complex has revealed a novel RNA binding mechanism. The apoprotein resembles a hinged container, similar to a clamshell, with an interior cavity large enough to contain an RNA sequence – in our case, 26 nts long. The structure is formed from six copies of the protein that are held together by the cap sequence, which is encoded in the middle of the protein. In the RNA-YicC complex, the clamshell has closed onto the RNA, with a set of arginine residues binding the RNA and exposing the hairpin to a set of glutamate residues that likely coordinate Mg^2+^ ions. This is a novel mechanism for RNA binding. Other endoribonucleases, such as those of the RNase III family, typically bind on the outside of an RNA structure.^14, 15^ The other RNase that binds RNA inside is PNPase, which forms a trimer as the active enzyme. However, in the case of PNPase, the RNA binds to a symmetric multimer, without much conformational change occurring upon binding.^16, 17^ YicC, on the other hand, binds asymmetrically and undergoes an substantial conformational change between apo and bound states. YicC, therefore, represents a new paradigm for protein binding of an RNA. Taking into account the unusual structural properties of YicC, we propose to name the enzyme RNase CS, for clamshell.

We do not understand how YicC cleaves twice across the hairpin, since the protein forms an asymmetric hexamer with a single active site. We can think of two possible mechanisms for this double cleavage. One model is that the hairpin is cleaved after position 3, and then the RNA rotates in the active site to cleave after position 14. Alternatively, the protein itself undergoes a conformational change to switch to the reverse asymmetric confirmation, exposing position 14 to the active site.

The result in Fig. 3D, comparing cleavage of a 26-mer and a 36-mer, suggests that even for an RNA substrate with a different sequence and likely different structure, YicC still cleaves at two sites, one 5’ proximal and one more distal. The trio of small, 5’ proximal products may indicate that the 36-mer substrate is not bound by the enzyme as well as the 26-mer, and therefore there is some heterogeneity in the actual cleavage site. In fact, fluorescence anisotropy experiments with YloC have shown that the 26-mer binds with higher affinity than the 36-mer (data not shown). In any event, these data allow us to hypothesize that the *in vivo* targets of YicC may be the 5’ end of RNAs that need to be degraded, and YicC serves to remove a protective 5’ end.

As we did here, Huang et al.^9^ also identified several arginine residues that contribute to RNA binding, including R30 and R280. Using a set of related RNA sequences as substrates, these authors reported that YicC recognizes primarily a GUG sequence. Our RNA substrates did not contain this trinucleotide sequence, but were nevertheless cleaved precisely at two sites by YicC. We note that our ribonuclease time-course assays, which contained a 1:30 protein:RNA ratio, were quite different from those of Huang et al., who examined only a single 30 min time point and used a 1:1 protein:RNA ratio.

Our results with YicC-family proteins from three organisms shows a strong conservation of endonuclease specificity (Fig. S6). The cleavage pattern from *E. coli* YicC and *B. subtilis* YloC is identical, despite these two organisms being at opposite ends of a bacterial phylogenetic tree^18^. The homology between these two proteins is relatively high. To demonstrate enzyme activity in a YicC-family protein a different source, we cloned and expressed the *B. burgdorferi* YicC, which has considerably less homology to *E. coli* YicC. In a phylogeny of bacterial ribonucleases (published before the discovery of YloC), *B. burgdorferi* sits about midway between *E. coli* and *B. subtilis* and has only half as many ribonucleases as the other two organisms.^18^ The *B. burgdorferi* YicC protein cleaved the 26-mer substrate RNA similarly, on either side of its hairpin structure, although yielding slightly different sized fragments from those generated by the *E. coli* and *B. subtilis* enzymes. The results with three YicC-family proteins from different locations on the bacterial phylogenetic tree suggest that YicC-family proteins from across the bacterial world will have endoribonuclease activity. Also, the two fluorescently labeled RNAs that were assayed here (Fig. 3D) showed a similar cleavage pattern, despite differences in RNA sequence and, most likely, structure. The capacity to accommodate and cleave different RNA molecules in a similar way may be a consequence of the clamshell-like ribonuclease mechanism.

While the function of YicC/RNase CS remains unknown, the conservation of the YicC family across all bacteria and, as we show here, the conserved biochemical nature of family members’ endonuclease activity, suggests an important function for this enzyme. Efforts are underway to identify native substrates of this novel family of ribonucleases.

## Methods

### Cloning, protein expression, and purification

For expression of the *B. burgdorferi* YicC protein, the 873-nt coding sequence was synthesized (Twist Biosciences) and amplified by PCR, followed by Gibson cloning into the modified pET47b vector used for the other YicC proteins.

For apo YicC purification for crystallography, we purified the protein as previously described. Briefly, an overnight culture of EG1261 was diluted 1:100 in 3L of LB medium containing 1 mM MgSO_4_, and the culture was grown to OD_600_ of 0.5. *YicC* expression was induced by addition of IPTG to 400 µM, followed by further incubation with shaking for three hours. Cell were washed with 20 mM Tris, pH 8.0, 250 mM NaCl and cell pellets were stored at −80°C. Cells were resuspended in TBS (20 mM Tris, pH 8.0, 150 mM NaCl). PMSF was added to 1 mM and lysozyme was added to 100 µg/ml. Cell resuspension was sonicated to lyse cells, and centrifuged at 36,000 × g for 30 min. The supernatant was loaded onto Ni-NTA resin (Qiagen) that was pre-equilibrated with TBS plus 25 mM imidazole. After 1 hour incubation, the resin was washed with TBS plus 50 mM imidazole and eluted with TBS plus 250 mM imidazole). After overnight cleavage by SUMO-protease, the target protein was loaded onto a Superdex 200 Increase Column (GE) in TBS pH 8.0, 150 mM NaCl. For Cryo-EM, the protein was purified the same way except after overnight cleavage by sumo-protease, the target protein was loaded onto a Hitrap Q column (GE) and eluted with a linear NaCl gradient, followed by size-exclusion chromatography (Superdex 200, GE) in buffer D (20 mM HEPES pH 7.5, 150 mM NaCl and 5 mM TCEP). Peak fractions were pooled, concentrated, flash frozen in liquid nitrogen and stored at −80°C. To assemble the RNA-YicC complex, the RNA was incubated with YicC protein at a molar ratio of 1.5:1 at 4°C for 40 min. Then the complex was loaded onto a Superdex 200 column equilibrated with buffer D for further purification.

### Purification of YicC protein for RNase and anisotropy assays

Cells were grown as described above. Frozen pellets from 100 ml of culture were resuspended in 15 ml ice cold TBS containing 250mM NaCl. PMSF was added to 1 mM and lysozyme was added to 100 µg/ml. The cell suspension was sonicated with a 1/8“ microtip (Qsonica) and amplitude of 45 for 12 minutes total processing time (15 sec bursts of sonication, followed by 45 sec cooling). Lysed cells were spun down at 36,000 × g for 30 min, and the supernatant was added to 0.25 ml of Ni-NTA and 250 mM imidazole was added for a final concentration of 25 mM imidazole. The lysate and resin was rotated for 1 hr at 4°C. The resin was washed with 20 ml of 50 mM imidazole in TBS/10% glycerol, and protein was eluted with 250 mM imidazole in TBS/10% glycerol. Four buffer exchanges with TBS/10% glycerol were performed in an Amicon-ultra 4 concentrator, to a final volume of less than 250 µl. Protein concentration was determined using the Bradford reagent (BioRad), and protein was stored at −80°C. Protein purity was examined by PAGE. YicC proteins were judged to be > 90% pure. For anisotropy experiments, the same protocol was scaled up for 2L cultures.

### Crystallography

After screening crystallization conditions, crystals were obtained and optimized by hanging drop vapor diffusion, using a reservoir containing 0.1M Tris pH 8.5, 0.3M lithium sulfate, and 25% PEG 3350. Data was collected at NSLSII beamline AMX, and was processed using autoproc ^19^. The data was anisotropic, so we used the autoproc anisotropic processing, which diffracted to 2.8Å in the best direction. The additional resolution from the anisotropic processing was critical in identifying sidechains. We then solved the structure by molecular replacement, using the AlphaFold structure of YicC as a search model.^20, 21^ The models were subsequently refined using Phenix ^22^ with rigid body refinement and multiple rounds of simulated annealing, minimization, atomic displacement parameter (ADP or B-factor) refinement and TLS refinement (determined using the TLSMD server) ^23, 24^ with interspersed manual adjustments using Coot ^25^. All structural figures were made with Pymol ^26^, except for the cryo-EM electron density figure which was made with Chimaera.^27^

### Electron microscopy

YicC-RNA complex sample at a concentration of 5 mg/ml was supplemented with 0.05% DDM detergent immediately before plunge-freezing to enable even distribution of particles on the grid. Then 3 µl of sample was applied to glow-discharged Quantifoil holey carbon grids (Cu, R1.2/1.3, 300 mesh). The grids were blotted for 2 s and plunged into liquid ethane with a Vitrobot plunger (4°C and 90% humidity). Cryo-EM data were collected with a Titan Krios microscope (FEI) operated at 300 kV and images were collected at a nominal magnification of 81,000 corresponding to a pixel size of 1.07 Å with a defocus range of −0.5 to −2 µm. The images were recorded on K3 electron direct detector in super-resolution mode at the end of a GIF-Quantum energy filter operated with a slit width of 15 eV. For data collection with a K3 detector, a dose rate of 15 electrons per pixel per second and an exposure time of 3.82 s were used, generating 70 video frames with a total dose of 60 electrons per Å^2^. The statistics for the cryo-EM data are listed in Table 2.

### Image processing

A total of 5,347 dose-fractionated videos of YicC-RNA complex were collected. The processing was done within cryoSPARC.^28, 29^ Motion correction was done by cryoSPARC’s Patch motion correction with an output F-crop factor of one-half. CTF estimation for each micrograph was calculated with Patch CTF estimation. Eight million particles were auto-picked and extracted from micrographs. Ab-initio models were generated as initial references for subsequent 3D classifications. Then the particles were sorted by multiple rounds of two-dimensional (2D) classification and three-dimensional (3D) classification to exclude bad particles; all classes containing YicC-RNA density were combined (342,211 particles) and used for NU-refinement, resulting in a final map at 3.22 Å resolution.

### RNase assay

YicC enzyme assays were performed in a 50 µl volume of RNase assay buffer, which was 5 mM MgCl_2_, 50 mM Tris pH 8.0, 7 mM NaCl, 100 mM KCl, and 400 µg/ml BSA. Unlabeled (20 pmoles) and IR-fluorescent-labeled RNA (10 pmoles) were used, for a final concentration of 600 nM. For fluorescent assay of 26-mer cleavage, unlabeled and labeled RNAs were DHB2051 and DHB2150 (Table S1). For the 36-mer, the unlabeled and labeled RNAs were DHB1866 and DH1879 (Table S1). YicC protein was added to a final concentration of 20 nM, giving a 1:30 protein:RNA ratio, except for the 36-mer and *B. burgdorferi* assays. At time points after addition of YicC, 10 µl of the reaction were removed into an equal volume Gel Loading Buffer II (Invitrogen) on ice to stop the reaction. Half of each sample (10 µl) was separated on a 20% denaturing polyacrylamide gel (Sequagel; National Diagnostics). Reaction products were visualized on a LI-COR Odyssey CLx imaging system and analyzed using LI-COR Image Studio software.

### Mass Spectrometry

After endonuclease digestion, the RNA was isolated by phenol-chloroform extraction before resuspension in 30% LC/MS grade methanol at approximately 1.25 micromolar concentration. After 10x dilution, 200 microliter RNA was loaded by a syringe pump for direct infusion. RNA was ionized by a heated electrospray ionization (HESI) probe and was analyzed with an Exploris 480 Orbitrap^™^ mass spectrometer (ThermoFisher). The flow rate was set at 10 microliter/min. The instrument was operated in the negative ionization mode at 3.0 kV for both MS and MS/MS acquisitions. The MS scan range was set at 500–6,000 m/z and the spectra were acquired at 240,000 resolution (at m/z = 200). Specific RNA precursor ions were selected for tandem MS (MS^2^) acquisition with an isolation window of 3 Th and maximum injection time of 100–800 ms. Different high-energy collisional dissociation (HCD) energies of 20%, 25% and 30% were evaluated to facilitate better fragmentation. After acquisition, the spectra were analyzed by a script from Ariadne (https://ariadne.riken.jp/index.html) and manually validated.

### Fluorescence Anisotropy

Cy3-labeled RNA (DHB1865; Table S3) was used at a fixed final concentration of 50 nM. All proteins were diluted twofold serially from 175 µM final concentration across 11 concentrations and a 12th at no protein, except for E281A protein which we diluted starting at 9 µM. After mixing protein in triplicate with RNA, the fluorescent polarization was read on a Victor NIVO plate reader with excitation at 545 nM, emission at 635 nM with a 595 nM dichroic mirror. The K_d_ values were calculated using Graphpad Prism and a log IC_50_ fit.

## Figures and Tables

**Table 1:** Data collection and refinement statistics (molecular replacement)

	YicC apo
**Data collection**	
Space group	P 1 21 1
Cell dimensions	
*a, b, c* (Å)	112.923 76.539 136.345
α, β, γ (°)	90 100.293 90
Resolution (Å)	35.19 – 2.80 (2.901 – 2.80)
*R*_sym_ or *R*_merge_	0.108 (1.101)
*R*_pim_	0.056 (0.520)
// σ/	8.8 (1.5)
Completeness spherical (%)	65.8 (11.2)
Completeness ellipsoidal (%)	93.0 (62.6)
Redundancy	4.6 (5.3)
CC(1/2)	0.996 (0.539)
**Refinement**	
Resolution (Å)	35.19 – 2.801 (2.901 – 2.801)
No. reflections	37484 (266)
*R*_work_ / *R*_free_	0.2264 / 0.2671
No. atoms	
Protein	13796
Ligand/ion	60
Water	
*B*-factors	
Protein	93.66
Ligand/ion	98.86
Water	
R.m.s. deviations	
Bond lengths (Å)	0.002
Bond angles (°)	0.39

*Data is from a single crystal. *Values in parentheses are for highest-resolution shell.

**Table 2:** Cryo-EM data collection, refinement and validation statistics

	YicC-RNA

**Data collection and processing**	

Magnification	81,000

Voltage (kV)	300

Electron exposure (e−/Å^2^)	50

Defocus range (μm)	0.5–2

Pixel size (Å)	1.07

Symmetry imposed	C1

Initial particle images (no.)	7,981,098

Final particle images (no.)	342,211

Map resolution (Å)	3.22

FSC threshold	0.143

Map resolution range (Å)	2.9–7.9

**Refinement**	

Initial model used (PDB code)	Apo structure

Model resolution (Å)	3.22 Å
FSC threshold	0.143

Map sharpening *B* factor (Å^2^)	-144.3

Model composition	
Non-hydrogen atoms	14326
Protein residues	13893
Nucleotides	431

*B* factors (Å^2^)	
Protein	
Nucleotides	

R.m.s. deviations	
Bond lengths (Å)	0.004
Bond angles (°)	0.394

Validation	
MolProbity score	1.21 (99th percentile)
Clashscore	4.26 (96th percentile)
Poor rotamers (%)	0%

Ramachandran plot	
Favored (%)	98.65%
Allowed (%)	1.35%
Disallowed (%)	0%
